# Comparative toxicity of synchrotron and conventional radiation therapy based on total and partial body irradiation in a murine model

**DOI:** 10.1038/s41598-018-30543-1

**Published:** 2018-08-13

**Authors:** Lloyd M. L. Smyth, Jacqueline F. Donoghue, Jessica A. Ventura, Jayde Livingstone, Tracy Bailey, Liam R. J. Day, Jeffrey C. Crosbie, Peter A. W. Rogers

**Affiliations:** 1 Department of Obstetrics and Gynaecology, University of Melbourne, Royal Women’s Hospital, Parkville, Victoria 3052 Australia; 2 School of Science, RMIT University, Melbourne, Victoria 3001 Australia; 3 Imaging and Medical Beamline, Australian Synchrotron, Clayton, Victoria 3168 Australia; 4 Australian Radiation Protection and Nuclear Safety Agency, Yallambie, Victoria 3085 Australia; 5 William Buckland Radiotherapy Centre, Alfred Hospital, Melbourne, Victoria 3004 Australia

## Abstract

Synchrotron radiation can facilitate novel radiation therapy modalities such as microbeam radiation therapy (MRT) and high dose-rate synchrotron broad-beam radiation therapy (SBBR). Both of these modalities have unique physical properties that could be exploited for an improved therapeutic effect. While pre-clinical studies report promising normal tissue sparing phenomena, systematic toxicity data are still required. Our objective was to characterise the toxicity of SBBR and MRT and to calculate equivalent doses of conventional radiation therapy (CRT). A dose-escalation study was performed on C57BLJ/6 mice using total body and partial body irradiations. Dose-response curves and TD_50_ values were subsequently calculated using PROBIT analysis. For SBBR at dose-rates of 37 to 41 Gy/s, we found no evidence of a normal tissue sparing effect relative to CRT. Our findings also show that the MRT valley dose, rather than the peak dose, best correlates with CRT doses for acute toxicity. Importantly, longer-term weight tracking of irradiated animals revealed more pronounced growth impairment following MRT compared to both SBBR and CRT. Overall, this study provides the first *in vivo* dose-equivalence data between MRT, SBBR and CRT and presents systematic toxicity data for a range of organs that can be used as a reference point for future pre-clinical work.

## Introduction

Advances in clinical radiation oncology over the past few decades have revolved around improving the conformity of dose-distributions to the tumour or modifying fractionation regimens to maximise the therapeutic ratio. More recently, the use of experimental radiation sources has led to novel radiobiological findings with potential clinical applications. Examples of this include remarkable normal-tissue sparing of the lung^1^ and brain^2^ following ultra-high dose-rate radiation therapy, known as a “FLASH” effect, and the spinal cord^3^ and brain^4,5^ when using micron scale spatially fractionated fields (microbeam radiation therapy; MRT). Given the demonstrated tumouricidal potential of these modalities^1,6,7^, their novel radiobiology could potentially be exploited for therapeutic benefit.

Radiation generated by a synchrotron source has the physical characteristics necessary to facilitate MRT and the potential to produce a FLASH normal tissue sparing effect using high dose-rate synchrotron broad-beam radiation therapy (SBBR). Both MRT and SBBR are being developed for future clinical use at the Imaging and Medical Beamline (IMBL) of the Australian Synchrotron. MRT is characterised by arrays of quasi-parallel micro-planar beams with a width of 25 to 100 µm that are typically spaced by 100 to 400 µm^8^. This arrangement allows for the delivery of in-beam (peak) doses that are at least an order of magnitude greater than the doses delivered between the beams (valley) due to scatter. In-beam dose-rates can exceed several hundred Gy/s. MRT exerts a differential effect on normal versus tumour tissue in regard to gene pathway modulation^9^, post-irradiation tissue repair^10^ and vascular architecture^11^. The mechanism behind the FLASH normal tissue sparing effect is yet to be determined, however, hypotheses include the differential activation of DNA damage pathways^1^ and the induction of transient hypoxia^1,12^. The oxygen depletion hypothesis is supported by *in vitro* data^13^ and *in vivo* experiments where transient radio-resistance was induced in mouse tails at high dose-rates^14^.

While there are a significant number of pre-clinical animal studies reporting on tissue responses to MRT, there is a lack of systematic dose-escalation data across a broad range of organs^15^. Currently, there is no published toxicity data for total body, abdominal or thoracic irradiation using MRT. Robust toxicity data for SBBR is similarly lacking. These are significant issues that must be addressed prior to a clinical trial.

Through a systematic dose-escalation study of conventional radiation therapy (CRT) versus MRT and SBBR using C56BLJ/6 mice, we provide the first *in vivo* report of dose-equivalence between these modalities. Dose-response curves for normal tissue toxicity were generated for each radiation modality following total body irradiation (TBI) and partial body irradiation (PBI) of the whole abdomen, head and thorax. The aim of this current study was to calculate TD_50_ values for each modality based on acute clinical endpoints related to weight and overall wellbeing, as a means of assessing dose-equivalence. We hypothesised that compared to CRT, there would be a normal tissue sparing effect using SBBR and that for synchrotron MRT, the valley dose would be the most important determinant of toxicity.

Biological methods of determining dose-equivalence between CRT and MRT are particularly insightful given the unique challenges of physically comparing a spatially homogenous field to an intrinsically inhomogeneous one. Endpoints such as clonogenic survival^16^, skin histopathology^17^, and normal tissue toxicity (current study), provide a measure of the gross effect of an entire array of microbeams compared to a homogenous field. Toxicity endpoints were chosen in this study given the fundamental need to determine the safety profile of both MRT and SBBR compared to CRT. Additionally, by reporting TD_50_ outcomes of TBI and PBI, we can compare MRT and SBBR to decades of classical radiobiology literature. Normal tissue toxicity data is essential to the planning of future pre-clinical animal studies and ultimately, for the selection of safe dose regimens in future clinical trials using MRT and/or SBBR^15^.

## Results

### Total Body Irradiation

**Figure 1:**
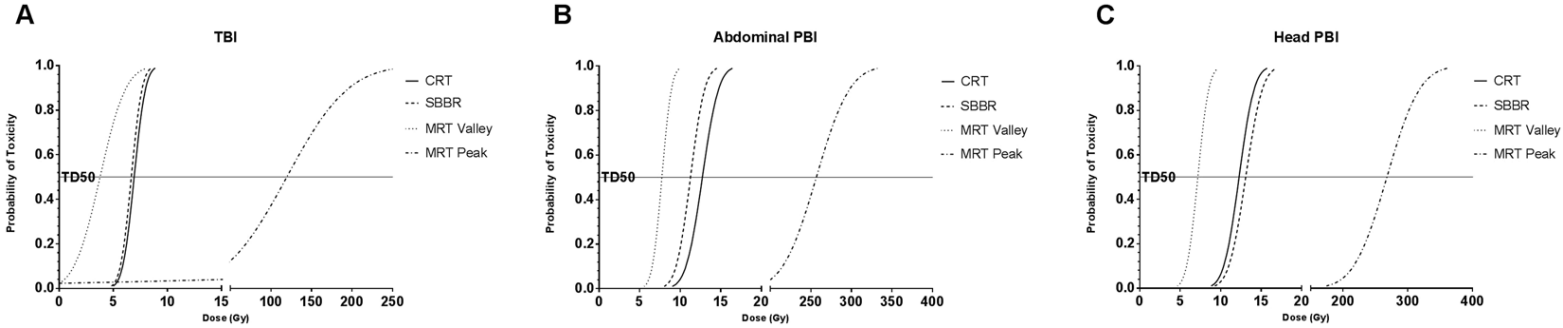
Dose response curves following conventional radiation therapy (CRT), microbeam radiation therapy (MRT) and high dose-rate synchrotron broad-beam radiation therapy (SBBR) for (**A**) total body irradiation (TBI), (**B**) abdominal partial body irradiation (PBI) and (**C**) head PBI. The horizontal TD_50_ line indicates the dose predicted to cause toxicity (>15–20% weight loss, severe diarrhoea, moribund behaviour) in 50% of the animals. For all three irradiation sites, there was no significant difference in TD_50_ values between CRT and SBBR. The peak MRT TD_50_ dose was an order of magnitude higher for each irradiation site. Dose response curves were generated using PROBIT analysis with N = 3–4 doses per modality and N = 4–5 mice per dose.

Mice in the two highest SBBR and CRT dose groups and the highest MRT dose group displayed signs of acute radiation syndrome, losing at least 15 to 20% body-weight within five to nine days following TBI. Several other animals in these groups showed delayed weight loss (at least 15 to 20%) and moribund behaviour, including lack of grooming, hunched posture and low activity levels between eleven and eighteen days following irradiation. The TD_50_ doses (Fig. 1A and Table 1) for these toxicities in the SBBR and CRT groups were 6.7 Gy (3.9–8.4 Gy) and 6.9 Gy, respectively. The MRT valley and peak TD_50_ doses were 3.8 (2.7–10.8) and 120 (84.3–343), respectively.

**Table 1:** Equivalent (TD_50_) doses for MRT, SBBR and CRT (Gy).

	*MRT-peak*	*MRT-valley*	*SBBR*	*CRT*
*TBI*	120 (84.3–343)	3.8 (2.7–10.8)	6.7 (3.9–8.4)	6.9
*Abdominal PBI*	257	7.7	11.3 (7.6–14.5)	12.7 (8.7–17.3)
*Head PBI*	268 (232–313)	7.2 (6.2–8.4)	13.1 (9.2–17.2)	12.3 (8.0–16.4)

TD_50_ doses calculated using PROBIT analysis^26^. 95% confidence intervals are stated in parentheses.

**Figure 2:**
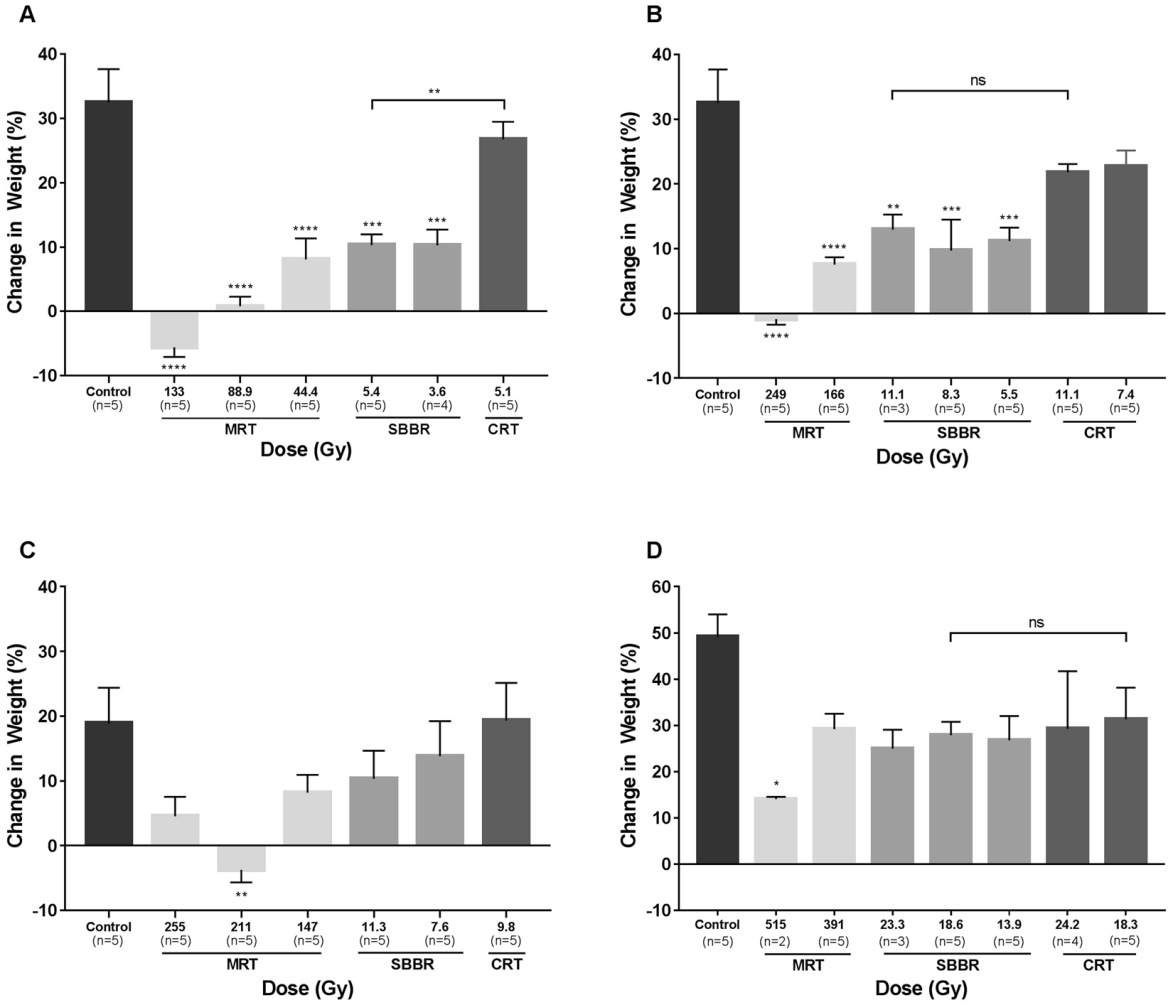
Post-irradiation weight gain for surviving mice. Weight gain is measured by a percentage change in weight compared to pre-experimental weight following (**A**) total body irradiation (TBI) 60 days post-irradiation, (**B**) abdominal partial body irradiation (PBI) 60 days post-irradiation, (**C**) head PBI 37 days post-irradiation and, (**D**) thoracic PBI 140 days following irradiation. Mice had subnormal weights compared to controls following irradiation regardless of the modality used or irradiation site. Mice in the high dose-rate synchrotron broad-beam radiation therapy (SBBR) and microbeam radiation therapy (MRT) groups had the most significant growth impairment. There was no statistically significant difference in weight gain between SBBR and conventional radiation therapy (CRT) at near equal doses, except for following TBI, with the 5.4 Gy SBBR group having significantly less weight gain compared to 5.1 Gy CRT. Differences between groups were analysed using ANOVA with N = 2–5 surviving mice per dose/modality; *p < 0.05, **p < 0.01, ***p < 0.001, ****p < 0.0001.

Surviving mice from all three irradiation modalities had sub-normal weights compared to control mice (Fig. 2A). All groups returned to their pre-experimental weight within 60 days of treatment, except the 133 Gy MRT group. These mice had a mean change in body weight of −5.6 ± 1.5% compared to their pre-experimental (baseline) weight (Fig. 2A).

### Abdominal PBI

**Figure 3:**
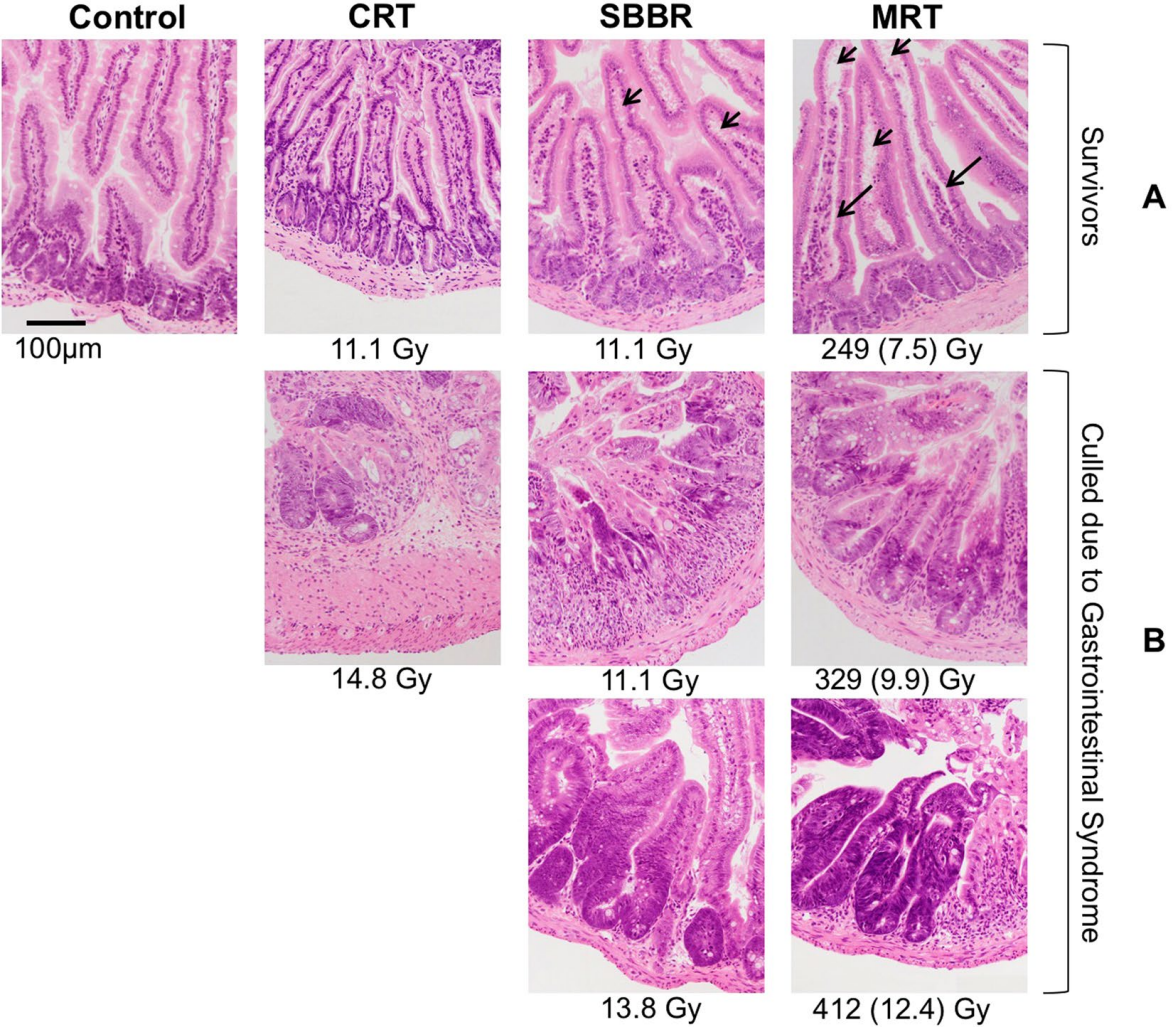
Intestinal histopathology following abdominal partial body irradiation. (**A**) Hematoxylin and eosin (HE) stained sections of small intestine from surviving mice showed relatively normal crypt villus architecture 60 days following irradiation with the exception of the 249 Gy microbeam radiation therapy (MRT) group. Short arrows point to regions where the sub-epithelial space has been extended and long arrows show where the epithelial cells of the villus have completely lifted off from the underlying lamina propria. For the MRT groups, valley doses are indicated in parentheses. (**B**) HE stained sections of small intestine from mice that were euthanized due to acute gastrointestinal syndrome showed a loss of normal crypt-villus architecture.

Mice receiving the two highest MRT and SBBR doses and the highest CRT dose displayed acute signs of gastro-intestinal syndrome, losing at least 15 to 20% body-weight and showing signs of severe diarrhoea, within five to six days of irradiation. The MRT peak and valley TD_50_ doses were 257 Gy and 7.7 Gy, and the SBBR and CRT TD_50_ doses were 11.3 Gy (7.6–14.5 Gy) and 12.7 Gy (8.7–17.3 Gy), respectively (Fig. 1B and Table 1). At necropsy, affected mice showed severe signs of dehydration and intestinal water retention, reflected histologically by the destruction of normal crypt-villus architecture and denudation of villi (Fig. 3B). No mice in the 166 Gy MRT group, 7.4 Gy CRT group or 8.3 and 5.5 Gy SBBR groups showed signs of acute gastrointestinal syndrome.

Surviving mice had sub-normal weights compared to control mice, regardless of the radiation modality used (Fig. 2B). All groups returned to their pre-experimental weight within 60 days of treatment except the 249 Gy MRT group, which had a mean change in body weight of −0.9 ± 0.9%. The gross crypt-villus architecture of surviving mice following irradiation was relatively normal, except for mice in the 249 Gy MRT group. The villi of these mice showed lifting of epithelial cells from the lamina propria layer and significant extension of the sub-epithelial space (Fig. 3A). The disruption of the epithelial-capillary interface in villi is consistent with abnormal mucosal absorption^18^ and could explain the subsequent growth impairment in the MRT groups.

### Head PBI

Mice in the two highest SBBR and CRT dose-groups, as well as the 255 Gy, 317 Gy and 377 Gy MRT groups, showed a decline in activity levels, grooming, appetite and water intake and/or 15 to 20% weight loss between seven to twelve days following irradiation. The TD_50_ values for these toxicities were 12.3 Gy (8.0–16.4 Gy) and 13.1 Gy (9.2–17.2 Gy) for CRT and SBBR, respectively. The MRT TD_50_ doses were 268 Gy (232–313 Gy) and 7.2 Gy (6.2–8.4 Gy) for the peak and valley, respectively (Fig. 1C and Table 1).

**Figure 4:**
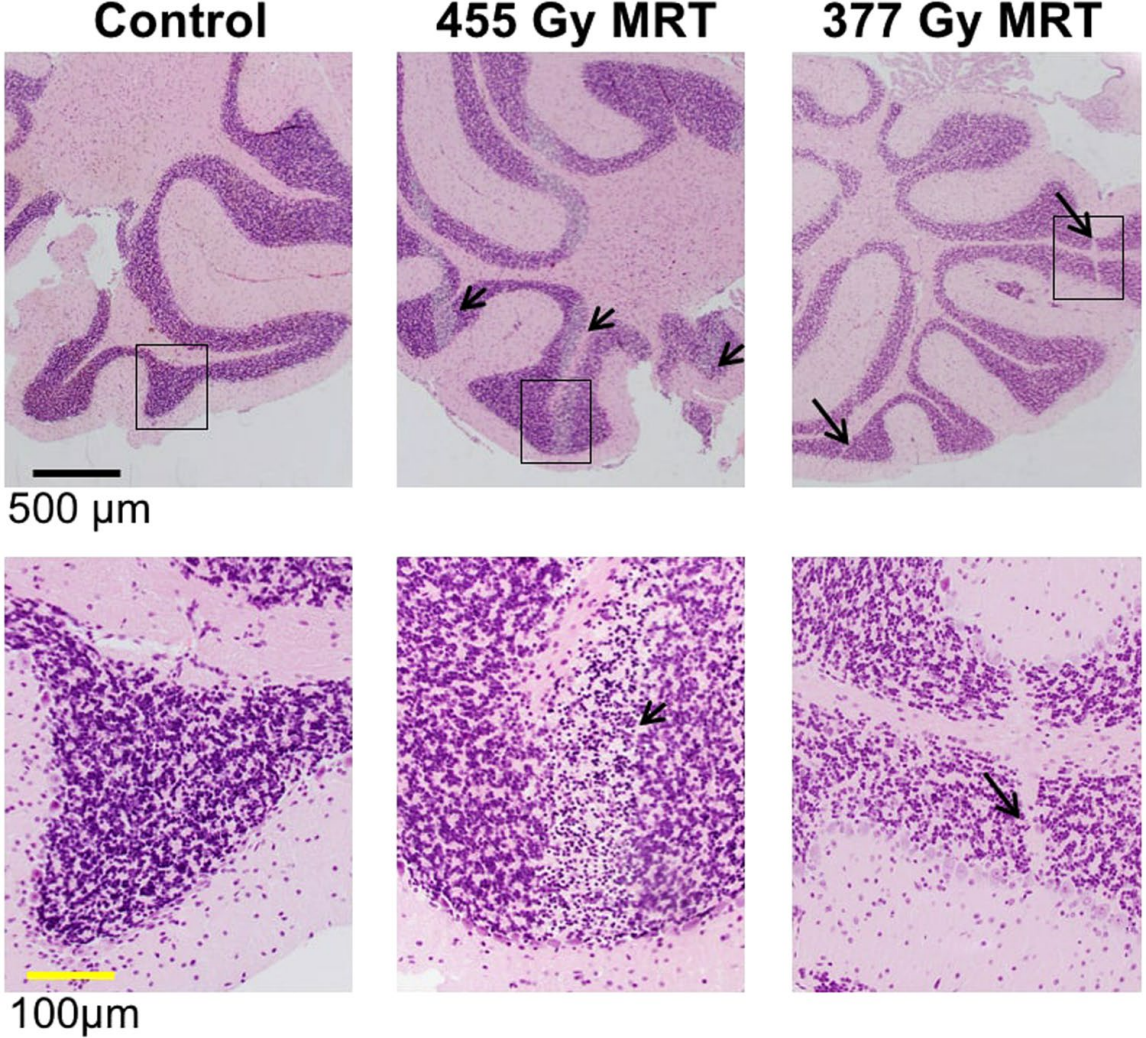
Histopathological changes to the cerebellum following microbeam radiation therapy (MRT). Top panels depict hematoxylin and eosin stained cerebellar sections and the lower panels depict magnified images corresponding to regions of interest (boxed). Wide, evenly spaced bands of cerebellar granular cell loss (short arrows), corresponding with microbeam paths, were evident following 455 Gy MRT and was associated with neurotoxicity within two to four hours of irradiation. Narrow bands of granular layer scarring (long arrows) were evident at 38 days following 377 Gy MRT in surviving mice.

In addition to weight loss and a moribund state, MRT caused neurological toxicities including ataxia, loss of balance and fitting within two to four hours at 455 Gy (5/5 mice) and 377 Gy (2/5 mice). These symptoms were not evident in the SBBR and CRT groups at the doses used in this study. Distinct and evenly spaced bands of cerebellar granular cell loss, corresponding with microbeam paths, were observed in mice that experienced acute neurological toxicities following MRT (Fig. 4). Cerebral and brainstem histology was unremarkable in these mice and these cerebellar changes were not observed in surviving mice from any other dose/modality. However, narrower bands of cerebellar scarring were observed in some long-term MRT survivors (Fig. 4), consistent with previous findings^19^.

Surviving mice in all irradiated groups had sub-normal weight gain compared to non-irradiated controls 37 days following irradiation, with the weight of mice in the 211 Gy MRT group 3.7 ± 1.9% below baseline (Fig. 2C). No signs of neurological toxicity or significant weight loss were observed in the 9.8 Gy CRT group or 7.6 and 11.3 Gy SBBR groups.

### Thoracic PBI

Several mice in the 587 Gy (4/5), 515 Gy (1/5) and 459 Gy (2/5) MRT groups experienced severe neurological toxicities within 2 to 4 hours of irradiation, including ataxia, loss of balance and fitting. These toxicities were unexpected and might be explained by collateral irradiation of upper spinal column during the thoracic irradiations. Further mice from the 587 Gy (1/5), 515 Gy (2/5) and 459 Gy (2/5) MRT groups presented with severe clinical symptoms including hunched posture, lack of grooming and poor body condition and were sacrificed due to 20% weight loss within six weeks of irradiation. Several mice from the 27.9 Gy SBBR (3/5), 24.2 Gy CRT (1/5) and 21.2 Gy CRT (2/5) groups presented with severe in-field cutaneous lesions within five weeks of irradiation and were sacrificed.

Remaining mice steadily gained weight following irradiation, albeit having sub-normal weight gain compared to non-irradiated control mice (Fig. 2D). Weight gain was significantly impaired in the 515 Gy MRT group relative to controls (p < 0.05) (Fig. 2D). Between 140 and 180 days after irradiation, n = 6 mice from CRT dose-groups and n = 1 mouse from the 23.3 Gy SBBR group began to show signs of cachexia. These mice were sacrificed following the loss of 20% body weight over the course of two weeks, with some mice presenting with low activity levels, laboured respiration, poor grooming and hunched posture.

**Figure 5:**
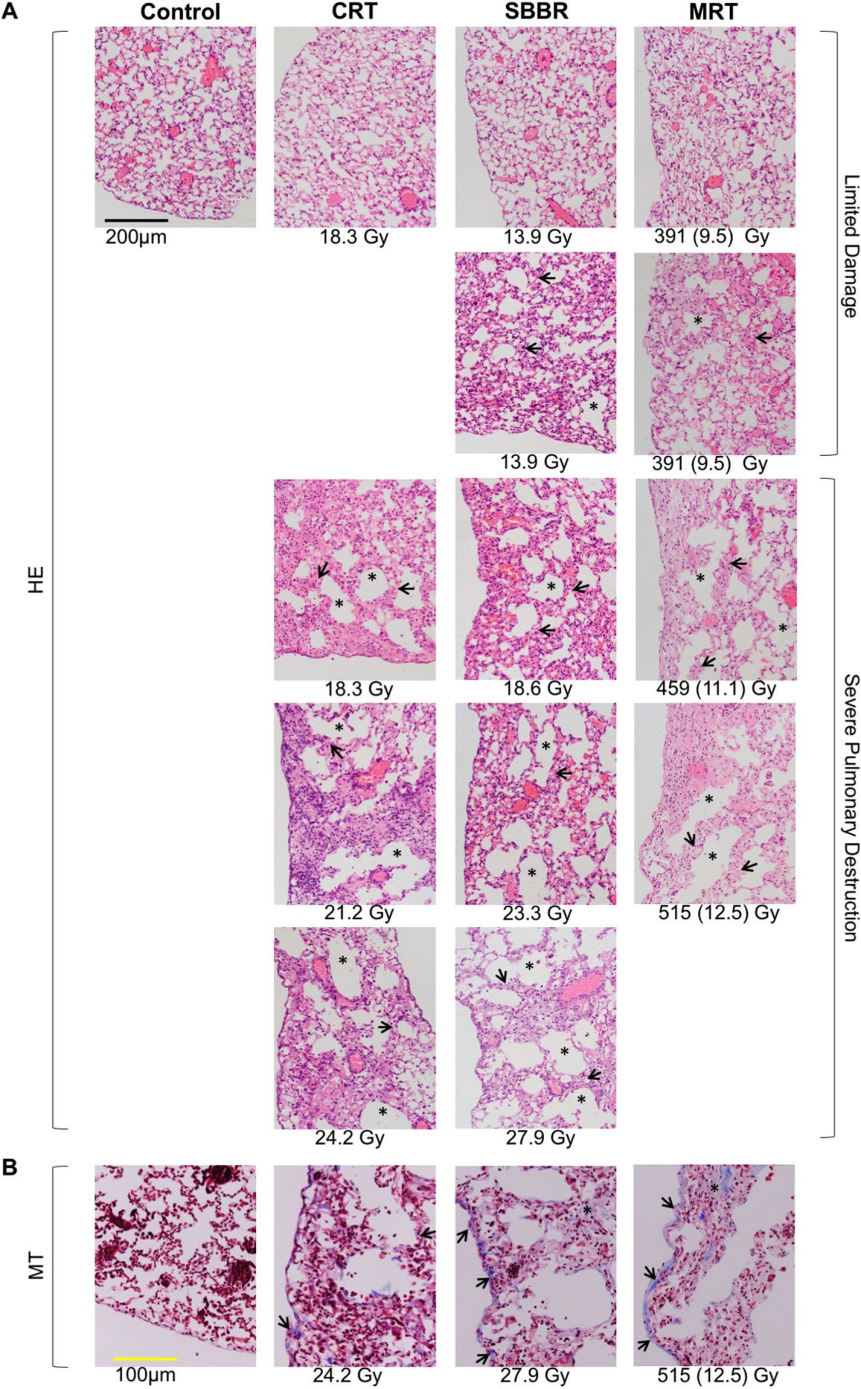
Pulmonary damage and fibrosis 170 to 180 days following thoracic partial body irradiation. (**A**) Hematoxylin and eosin stained lung sections showed severe pulmonary damage, including alveolar destruction, airspace enlargement (asterisks) and the thickening of alveolar walls (short arrows), in all dose groups for each modality. For the microbeam radiation therapy groups, valley doses are indicated in parentheses. (**B**) Masson’s Trichrome staining revealed the deposition of collagen fibres (blue) in sub-pleural (short arrows) and intra-parenchymal (asterisks) regions.

The lungs of surviving mice from each radiation modality showed signs of inflammation and long-term pulmonary destruction 170 to 180 days following irradiation with the most severe damage evident at the highest doses of each modality. For every dose and modality, histopathology revealed alveolar destruction, airspace enlargement and the thickening of alveolar walls (Fig. 5A). Masson’s Trichrome stained sections confirmed the presence of fibrosis, with collagen deposition in sub-pleural and intra-parenchymal regions (Fig. 5B). PROBIT analysis was not performed on the thoracic PBI series due to the premature sacrifice of mice from all three irradiation modalities.

## Discussion

To our knowledge, this is the first *in vivo* study reporting dose-equivalence between MRT, SBBR and CRT. Tissue specific TD_50_ values were calculated for each modality. There was no gender bias specific to the toxicities observed across the TBI and PBI experiments. Key findings of this study include: (1) no clear evidence of a normal tissue sparing effect using a SBBR dose-rate of 37 to 41 Gy/s, (2) the valley MRT dose as the most relevant parameter for acute toxicity, and, (3) long-term detrimental effects of MRT on growth, despite the acute tolerance of irradiation.

Importantly, the TD_50_ values for TBI and abdominal PBI calculated for CRT in our study were comparable to LD_50_ values previously reported by Booth *et al*.^20^. These data were based upon similar levels of toxicity in the same mouse model and at a comparable dose rate of 0.01 Gy/s. Furthermore, Favaudon *et al*.^1^ report pulmonary fibrosis at doses higher than 15 Gy when using a conventional dose-rate of 0.03 Gy/s, consistent with our findings. There is no comparable data for head PBI previously reported in the literature to validate the CRT group in our study.

Our TD_50_ data provides no clear evidence for a normal tissue-sparing effect using SBBR, and at the doses used for thoracic PBI, SBBR was not protective against destructive pulmonary changes relative to CRT. The protective effect of FLASH radiation therapy has previously been shown to be dose-rate dependent^2^. Following whole brain irradiation, the memory of C57BLJ/6 mice was preserved when 10 Gy was delivered at 100 Gy/s but was significantly impaired relative to non-irradiated controls with a dose-rate of 60 Gy/s or less^2^. A significant decline in memory function was also observed when the dose-rate was reduced from 60 Gy/s to 30 Gy/s^2^. Furthermore, lung-sparing benefits of FLASH radiation therapy are reported at dose-rates of 60 Gy/s^1^. It is therefore possible that the SBBR dose-rates of approximately 37 to 41 Gy/s used in our study were too low to elicit a definitive normal tissue sparing effect compared to CRT. Although Favaudon *et al*.^1^ and Montay-Gruel *et al*.^2^ delivered FLASH radiation therapy using electron radiation rather than photons, there was no difference in lung fibrogenesis when comparing electrons versus gamma-ray photons at a conventional dose rate^1^. Furthermore, given that x-rays, gamma-rays and electrons are all examples of sparsely ionizing radiation and have a comparable relative biological effectiveness^21^, it is unlikely that radiation quality influenced the SBBR toxicity outcomes presented in our study or those reported previously^1,2^.

The TD_50_ values for CRT correlated more closely with the MRT valley dose that the MRT peak dose across the TBI and PBI experiments. This supported our hypothesis that the valley dose was the most useful parameter for comparing MRT and CRT with regards to acute normal tissue toxicity. However, the TD_50_ values for the MRT valley were still substantially lower than the CRT TD_50_ doses, suggesting that the peak and intermediate doses delivered by MRT still influence acute toxicity. Further to this, it is apparent from our head PBI series that acute neurological toxicity is possible within hours of MRT irradiation. This is most likely due to high peak doses, rather than the valley doses, given that the corresponding valley doses in the affected MRT groups were markedly lower than the SBBR and CRT doses that were tolerated without acute neurotoxicity.

Histological analysis demonstrated a marked loss of granular cells in the cerebellum of MRT-irradiated mice that experienced acute neurological toxicities. This loss of cells directly correlated with the microbeam paths. The cerebellum has been previously shown to tolerate peak MRT doses in the order of 200 Gy without significant signs of long-term neurological or developmental difficulties^19^, which is much lower than the highest MRT doses used in this study. To our knowledge, our results provide the first report of acute neurological symptoms within hours of MRT and suggest that the cerebellum should be considered separately to the cerebrum as an organ at risk in future studies. Mukumoto *et al*.^22^ reported an LD_50_ of 600 Gy for MRT (peak dose) following whole brain irradiation of C57BLJ/6 mice, compared to a TD_50_ of 268 Gy in this study. Aside from assessing lethality rather than toxicity, the significantly higher LD_50_ reported by Mukumoto *et al*.^22^ could be explained by a number of factors including the use of narrower microbeams (25 µm versus 50 µm) and a smaller field size.

Finally, while spatial fractionation in MRT enables the delivery and acute tolerance of high doses of radiation, the long-term effects on growth could be profound. We consistently observed greater growth impairment following MRT for TBI and PBI compared to SBBR and CRT. For abdominal PBI, this was associated with late morphological changes to the intestinal mucosa. Increasing the dose delivered per fraction is classically considered to increase the risk of late irradiation damage^23^. As such, hypofractionated treatment regimens (greater than 2 Gy per fraction) are used in the clinic with a high degree of selectivity. Our data suggests that similar caution should be taken when using high peak MRT doses and that the radiobiology of tumours and surrounding normal tissue should be considered when determining possible treatment sites for the future use of MRT. However, it is important to acknowledge that while the TBI and PBI irradiations used in this study provide fundamental toxicity data, they are not representative of the conformal CRT treatments typically delivered in the clinic today which minimise the exposure of organs at risk to radiation. This limitation is important given the relationship between irradiation volume and normal tissue toxicity^24,25^.

There are limitations of our experimental protocol that should be noted. The dose-rates of the MRT groups were in the order of 300 Gy/s. However, it still took several seconds to move the mouse vertically through the synchrotron x-ray beam for TBI and PBI. Thus, it is possible there will be effects of both organ motion (e.g. cardiac pulsation) and gross changes in animal position during these irradiations. These movements may lead to some blurring or smearing of the peak-valley MRT dose distribution, in effect, exaggerating the physical microbeam width. In addition, the lead shields placed on the plastic mouse holder contributed to some scattered x-ray dose.

The effects of animal motion might be evident in the 455 Gy PBI head group (Fig. 4), where the observed band of cerebellar granular cell damage was closer to 100 μm in width rather than 50 μm. Additionally, there is a lateral penumbra directly on either side of each microbeam, which represents regions of dose intermediate between the peak and valley dose. The penumbra could also explain why biological bands of microbeam damage appear wider than the physical microbeams.

Undoubtedly, refinements could be made to our experimental protocol in future studies, but these should not detract from the important data we report on organ toxicity, particularly for the gut and lung. Finally, a notable limitation of our TD_50_ data is the relatively small sample size per group for each radiation modality and dose. This restriction on group size was necessary to satisfy animal ethics requirements, given the expectation and manifestation of severe radiation-induced toxicities.

To further investigate the radiobiological differences between SBBR, MRT and CRT at a molecular level, and to histologically quantify radiation-induced damage, future *in vivo* studies using the median toxicity doses calculated in this study are planned. Furthermore, these toxicity-based dose-equivalence data will be used in pre-clinical studies of tumour-bearing mice to investigate whether an improved therapeutic ratio can be achieved using MRT or SBBR compared to CRT.

To conclude, the observations made in this current dose-equivalence study provide an important step towards understanding the relative toxicity of SBBR and MRT compared to CRT. We did not observe a FLASH normal tissue sparing effect at the SBBR dose-rates used in this study. We report pulmonary and gastrointestinal toxicity data for MRT for the first time and demonstrate that the MRT valley dose is a better predictor of acute toxicity than the peak dose. Importantly, we also report long-term growth impairment following MRT. The dose-response curves and toxicity data generated in this study will provide a reference point for future *in vivo* studies. These studies should aim to identify scenarios where the potential radiobiological advantages of SBBR and MRT can be best exploited for an enhanced therapeutic effect.

## Methods

### Irradiation Sources

**Figure 6:**
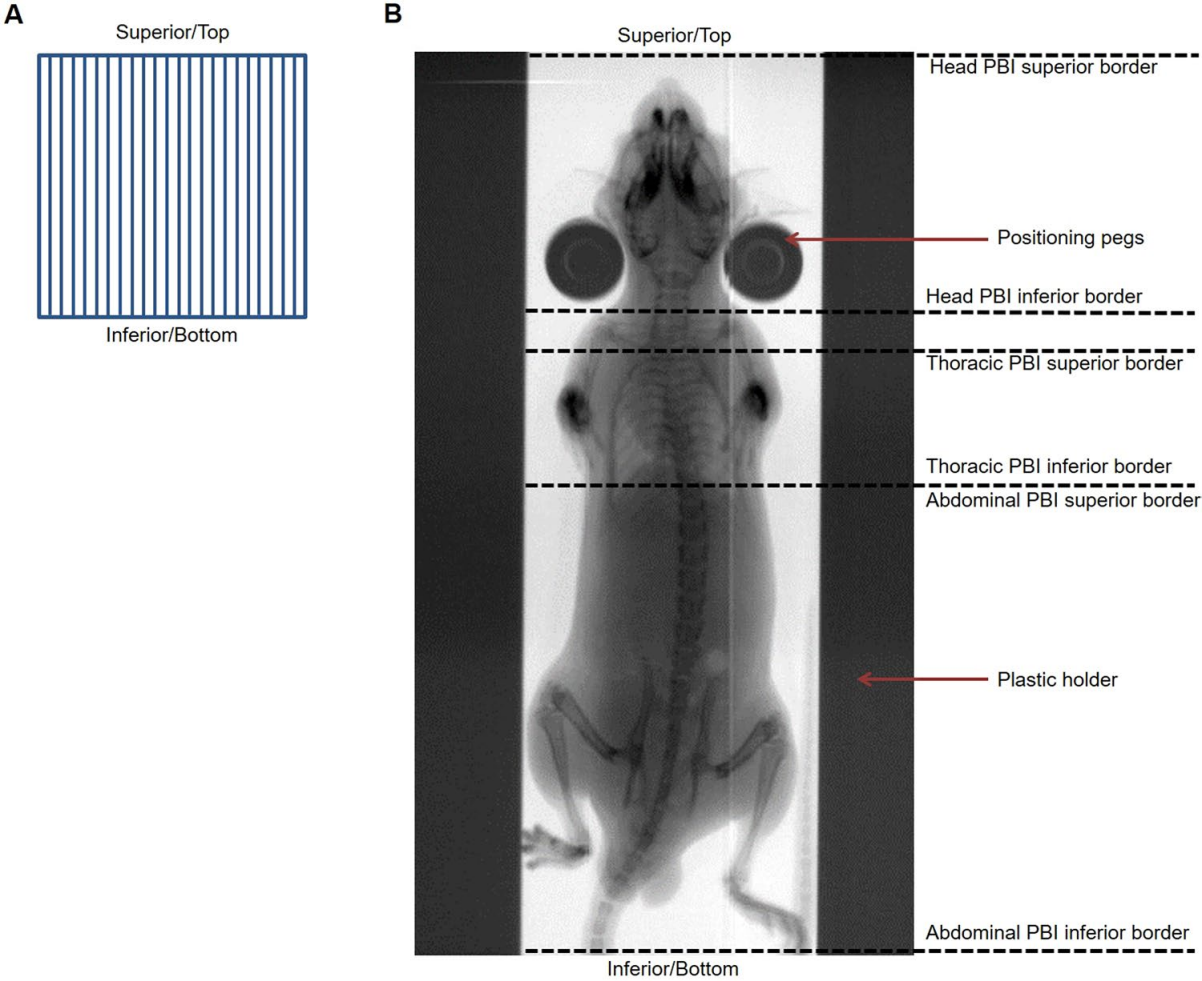
Mouse positioning for irradiation. (**A**) For microbeam radiation therapy at the Imaging and Medical Beamline, microbeams were orientated vertically and therefore in parallel with respect to the superior-inferior plane of mice. Microbeam width and spacing are not to scale in this beams-eye-view diagram. (**B**) Radiographic imaging shows mice positioned vertically in the path of radiation. Mice were gently strapped to a plastic holder, with support provided by small positioning pegs. Superior and inferior field borders for the partial body irradiations are denoted by overlayed dotted lines.

MRT was performed at the IMBL at the Australian Synchrotron using an array of vertically orientated quasi-parallel microbeams (Fig. 6A) with a width of 50 µm and centre-to-centre spacing of 400 µm. The in-beam dose-rate was between 276 and 319 Gy/s and the mean photon energy was 95 keV. SBBR was also performed at the IMBL at a dose-rate between 37 and 41 Gy/s with a mean photon energy of 124 keV. CRT was delivered using a Comet MXR-320/26X-ray tube with Gulmay GX320 generator at the Australian Radiation Protection and Nuclear Safety Agency using a dose-rate of 0.05 to 0.06 Gy/s. The effective CRT photon energy was 93 keV.

Monte Carlo generated percentage depth-dose curves for MRT, SBBR and CRT are presented in Supplementary Fig. 1. The relative change in dose-deposition with depth is almost identical for each of the three modalities within the first 30 mm, therefore making any dosimetric differences negligible in the context of the size of a mouse.

### Dosimetry and Field Placement

The dose, dose-rate and field size for each type of irradiation is specified in Table 2. Mice were positioned in a plastic holder, held vertically in the radiation path (Fig. 6B) and irradiated in an anterior to posterior direction. For abdominal PBI, the radiation field encompassed the entire abdomen and pelvis with the superior field border at level of the xyphoid process of the sternum (Fig. 6B). Head PBI included the entire brain, brainstem and superior part of the cervical spinal cord (Fig. 6B). During thoracic PBI, both lungs and the heart were irradiated, with the inferior field border at the xyphoid process of the sternum to avoid collateral irradiation of the abdomen (Fig. 6B). Lead shields were used to achieve the required field borders.

**Table 2:** Dose groups, field sizes and dose-rates for total and partial body irradiations.

*MRT Peak* (*Valley*) (*Gy*)	*SBBR* (*Gy*)	*CRT* (*Gy*)
**TBI**		
30 *mm* × 100 *mm*	30 *mm* × 100 *mm*	100 *mm diameter circle*
*Dose-rate*: 291 *Gy*/*s*	*Dose-rate*: 39.1 *Gy*/*s*	*Dose-rate*: 0.05 *Gy*/*s*
44.4 (1.4)	3.6	5.1
59.1 (1.9)	5.4	7.6
88.9 (2.8)	7.2	10.1
133 (4.2)	9.0	
**Abdomen PBI**		
30 *mm* × 60 *mm*	30 *mm* × 60 *mm*	100 *mm* × 60 *mm*
*Dose-rate*: 288 *Gy*/*s*	*Dose-rate*: 38.3 *Gy*/*s*	*Dose-rate*: 0.06 *Gy*/*s*
166 (5.0)	5.5	7.4
249 (7.5)	8.3	11.1
329 (9.9)	11.1	14.8
412 (12.4)	13.8	
**Head PBI**		
30 *mm* × 30 *mm*	30 *mm* × 30 *mm*	100 *mm* × 30 *mm*
*Dose-rate*: 280 *Gy*/*s*	*Dose-rate*: 41.3 *Gy*/*s*	*Dose-rate*: 0.06 *Gy*/*s*
147 (3.9)	7.6	9.8
178 (4.8)	11.3	14.7
211 (5.7)	15.1	19.6
255 (6.8)	18.9	
***Dose-rate*** **: 319 ** ***Gy*** **/** ***s***		
317 (8.5)		
377 (10.1)		
455 (12.2)		
**Thoracic PBI**		
30 *mm* × 20 *mm*	30 *mm* × 20 *mm*	100 *mm* × 20 *mm*
*Dose-rate*: 276 *Gy*/*s*	*Dose-rate*: 36.8 *Gy*/*s*	*Dose-rate*: 0.06 *Gy*/*s*
391 (9.5)	13.9	18.1
459 (11.1)	18.6	21.2
515 (12.5)	23.3	24.2
587 (14.2)	27.9	

All doses were prescribed to a depth of 5 mm in water. A full description of our dosimetry protocol for all three irradiation modalities is provided in a Supplementary Methods section. In short, SBBR and CRT dosimetry is based on full-scatter reference conditions as previously described^26–28^ with Monte Carlo simulations used to adjust for the loss of backscatter due to the plastic mouse holder. The theoretical phantom used to mimic the scatter conditions of a mouse in the plastic mouse holder is shown in Supplementary Fig. 2. MRT peak doses were derived from the SBBR dosimetry^29^ and Monte Carlo simulations of the peak-to-valley dose ratio (PVDR) used to calculate the valley doses. The PVDR at 5 mm depth for the MRT irradiations was between 31.8 (TBI) and 41.3 (Thoracic PBI). The total uncertainty (k = 1) for the irradiations are as follows; CRT – 6.1%, MRT peak – 5.1%, MRT valley – 8.6%, SBBR – 4.8%. A detailed uncertainty budget is available as Supplementary Data.

### Mice and Ethics Statement

A total of 235 male and female C57BLJ/6 mice aged 8 to 10 weeks old at irradiation were purchased from the Monash Animal Research Platform and housed at the animal facilities of the Australian Synchrotron and Royal Melbourne Hospital. Animal procedures were approved by the University of Melbourne Office for Research Ethics and Integrity (ethics identification no. 1613833) and performed in accordance with relevant guidelines and regulations.

Mice in the TBI, abdominal PBI and head PBI groups were monitored at least once per day post-irradiation and euthanized according to strict intervention criteria. Mice receiving thoracic PBI were monitored twice per week following irradiation. Specific toxicity endpoints were severe (20%) weight loss compared to pre-experimental weight, 15% weight loss and signs of poor well-being (severe diarrhoea, moribund behaviour, hunched posture, lack of grooming) and abnormal neurological signs (seizures, fitting, balance disorders). Growth following irradiation was assessed by calculating the percentage change in weight of each mouse at a given time-point compared to baseline, which was defined as the weight immediately prior to irradiation.

### Histopathology

Mice were humanely euthanized and tissue was collected for each mouse once reaching one of the aforementioned toxicity endpoints or at the end of the experimental post-irradiation follow-up period (60 to 68 days for TBI and abdominal PBI, 38 days for head PBI and 170 to 180 days for thoracic PBI). The intestinal tract and brain were harvested and fixed in formalin for 24 hours. Lungs were gently inflation-fixed using formalin and post-fixed in formalin for 24 hours. After fixation, tissue was embedded in paraffin and 4 µm thick sections were stained with hematoxylin-eosin reagent for analysis. Lung sections were additionally stained with Masson’s Trichrome. Slides were viewed using an Olympus IX83 wide-field microscope (Olympus Corp., Tokyo, Japan) and images were taken using an Olympus DP22 colour camera (Olympus Corp., Tokyo, Japan).

### Statistics

N = 5 mice were irradiated per dose group for each modality. PROBIT analysis^30^ was performed using the IBM SPSS Statistics suite (version 24) (IBM Corp., Armonk, NY, USA) to model the probability of toxicity as a function of radiation dose. The TD_50_ dose, which was the dose associated with a 50% incidence of a specified toxicity, was calculated for each radiation modality with a 95% confidence interval stated in parentheses where possible. Weight data was analysed using a one-way analysis of variance (ANOVA) in GraphPad Prism version 7.0 (GraphPad Software Inc, San Diego, CA, USA) with a significance level of 0.05. These data are presented as a mean percentage change compared to baseline (pre-experimental weight) ±s.e.m; *p < 0.05; **p < 0.01; ***p < 0.001; ****p < 0.0001; ns, not statistically significant.

## Electronic supplementary material

Supplementary Information

## Data Availability

All datasets generated and analysed during this study are available from the corresponding author upon reasonable request.
