# Supplementary material for: Comparative toxicity of synchrotron and conventional radiation therapy based on total and partial body irradiation in a murine model
Source: Sci Rep. 2018 Aug 13;8:12044. doi: 10.1038/s41598-018-30543-1 (PMC6089899; doi:10.1038/s41598-018-30543-1)
Supplement: Supplementary file 1 — Supplementary Information [file 41598_2018_30543_MOESM1_ESM.pdf]

Comparative toxicity of synchrotron and conventional radiation therapy based on total and partial body irradiation in a murine model

Lloyd M. L. Smyth[1], Jacqueline F. Donoghue[1,2], Jessica A. Ventura[1], Jayde Livingstone[3], Tracy Bailey[4], Liam R. J. Day[2], Jeffrey C. Crosbie[2,5] and Peter A. W. Rogers[1,*]

[1]*Department of Obstetrics and Gynaecology, University of Melbourne, Royal Women's Hospital, Parkville, Victoria 3052, Australia*

[2]*School of Science, RMIT University, Melbourne, Victoria 3001, Australia;*

[3]*Imaging and Medical Beamline, Australian Synchrotron, Clayton, Victoria 3168, Australia*

[4]*Australian Radiation Protection and Nuclear Safety Agency, Yallambie, Victoria 3085, Australia*

[5]*William Buckland Radiotherapy Centre, Alfred Hospital, Melbourne, Victoria 3004, Australia.*

*parogers@unimelb.edu.au

Supplementary Information

Further explanation of dosimetry method, traceability and uncertainty

In all three delivery modes (SBBR, MRT and CRT), the absorbed dose to water was determined at a point on the beam axis at a depth of 5 mm in a theoretical phantom designed to mimic the scatter conditions of a mouse in the custom plastic (poly-methyl-methacrylate; PMMA) holder used during the irradiations (Supplementary Fig. 2). In this phantom the mouse is modelled as a rectangular water slab (2 cm wide x 10 cm high x 1.5 cm thick) with a 0.5 cm air gap, and PMMA holder of 7 cm wide x 16 long and 2.4 cm thick. While this model does not take into account any details of the mouse itself, it does include the overall scatter conditions, and therefore is a more accurate choice than using the dose in a full-scatter water phantom, or the incident air kerma. Initially the phantom calculations were performed to estimate the uncertainty in using the full-scatter water dose as a surrogate for the mouse dose. However, when the magnitude of the difference between full-scatter water and the mouse phantom became apparent, it became clear that this was a better surrogate for the actual mouse dose. Importantly, the mouse plastic phantom (with a lack of backscatter) has a pronounced effect on MRT valley dose compared to the full-scatter water phantom.

SBBR

Dosimetry for SBBR was performed using a PTW (Freiburg, Germany) model 31014 pinpoint ionization chamber in a virtual water phantom according to the protocols previously described by Lye et al.[1] and Livingstone et al[2]. The chamber was calibrated by the National Measurement Institute of Germany, Physikalisch-Technische Bundesanstalt (PTB), for absorbed dose to water in conventional X-ray beams, and the calibration coefficient was interpolated in HVL to the synchrotron beam spectrum to obtain $N_{D,w}$ = 2.70 x 10^9 Gy/C with an uncertainty of 2.2% (k=1). We estimate an additional 0.5% uncertainty arises from the difference in the field size, depth and spectrum of the synchrotron beam compared to the PTB calibration beams. Charge from the Pinpoint chamber was integrated as it and the phantom were scanned through the 1 mm high x 30 mm wide synchrotron beam to deliver a uniform rectangular field. After correction for recombination, the dose at the

measurement depth (20 mm) was multiplied by a measured depth-dose relationship (PDD) to obtain the dose at 5 mm depth. Dosimetry was performed for the four field sizes (30 mm x 20 mm, 30 mm x 30 mm, 30 mm x 60 mm, 30 mm x 100 mm) which produce slightly different doses due to increased backscatter in the larger fields. In this dynamic delivery mode, the incident air kerma and beam size is constant. The dose is controlled by the velocity of the sample as it is scanned through the beam. Uncertainties in all of these factors give rise to a combined uncertainty in the delivered dose at 5 mm depth in a water phantom of 2.4% (k=1). An additional uncertainty due to air around the mouse and loss of backscatter in the mouse phantom was calculated from Monte Carlo simulations to be in the range of 0.94-0.90 leading to combined uncertainties of 4.8 % for SBBR.

MRT

Dosimetry for MRT was determined relative to the measurements made for SBBR. Previous work by Poole et al.[3] used GEANT4 Monte Carlo models to establish the output factor (OF) for the peak dose and the peak to valley dose ratio (PVDR). The OF is the ratio of the dose in the peaks to the dose in the broad beam when the MRT collimator is removed (i.e. the SBBR case). The PVDR is the ratio of the dose in the peaks to the dose in the valleys. In the current study, the PVDR varied from 31.8 (TBI) to 41.3 (Thoracic PBI). The uncertainty in the OF is estimated to be 1.7% and in the PVDR is 6.9% (k=1). These uncertainties are added in quadrature with the SBBR uncertainty to obtain a combined uncertainty of 5.1% for the peak doses and 8.6% for the valley doses in MRT.

CRT

Dosimetry for CRT was performed with an IBA (Schwarzenbruck, Germany) FC65-G ionization chamber in air without its build-up cap. The chamber was calibrated by the Australian Radiation Protection and Nuclear Safety Agency (ARPANSA) for air kerma, and absorbed dose to water at the surface of a full-scatter water phantom was calculated following the American Association of Physicists in Medicine Task Group 61 protocol[4], for the largest field size (approximately 10 cm diameter). A PDD from Supplement 25 of the British Journal of Radiology[5] was applied to calculate dose at 5 mm depth, and Monte Carlo calculations were performed to account for the loss of backscatter during the mouse irradiations. These calculations included the

beam size for each case, replaced the full-scatter water phantom with a model of the PMMA mouse holder and, for the PBI cases, the Pb shields. The mouse itself was modelled as a rectangular slab of water with a 5 mm air gap surrounding it (Supplementary Fig. 2). The combined D_W uncertainty at 5mm depth was calculated to be 6.1% for CRT. The full uncertainty budget for CRT, SBBR and MRT is included as a Supplementary Dataset.

References

1. Lye, J. E. *et al.* Absolute dosimetry on a dynamically scanned sample for synchrotron radiotherapy using graphite calorimetry and ionization chambers. *Physics in Medicine & Biology* **61**, 4201 (2016).
2. Livingstone, J. *et al.* Preclinical radiotherapy at the Australian Synchrotron's Imaging and Medical Beamline: instrumentation, dosimetry and a small-animal feasibility study. *Journal of synchrotron radiation* **24**, 854-865 (2017).
3. Poole, C. M., Day, L. R., Rogers, P. A. & Crosbie, J. C. Synchrotron microbeam radiotherapy in a commercially available treatment planning system. *Biomed. Phys. Eng. Express* **3**, 025001 (2017)
4. Ma, C. M. *et al.* AAPM protocol for 40-300 kV x-ray beam dosimetry in radiotherapy and radiobiology. *Med. Phys.* **28**(6), 868-893 (2001).
5. British Institute of Radiology, *Central axis depth dose data for use in radiotherapy departments*. Suppl. 25 (British Institute of Radiology, London 1996).

Supplementary Figures

Percentage depth dose curves – all modalities

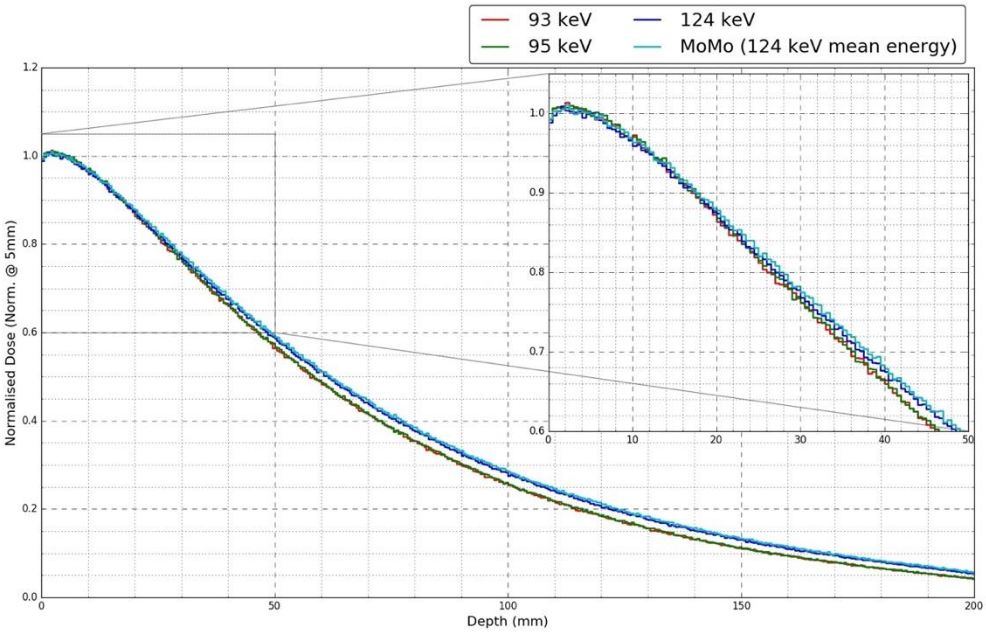

Supplementary Fig. 1. Monte Carlo (GEANT4) generated percentage depth dose (PDD) curves for keV x-ray beams incident on a water phantom. Curves are for mono-energetic x-ray beams representing microbeam radiation therapy (MRT; 95 keV), ultra-high dose rate radiation therapy (SBBR; 124 keV) and conventional radiation therapy (CRT; 93 keV). The Mo-Mo curve represents a beam with a spectrum of x-ray energies and a mean energy of 124 keV (SBBR). These PDD plots show that the relative change in dose deposition with depth is almost identical for the first 30 mm (> thickness of a mouse) for all three modalities.

Plastic mouse-holder geometry

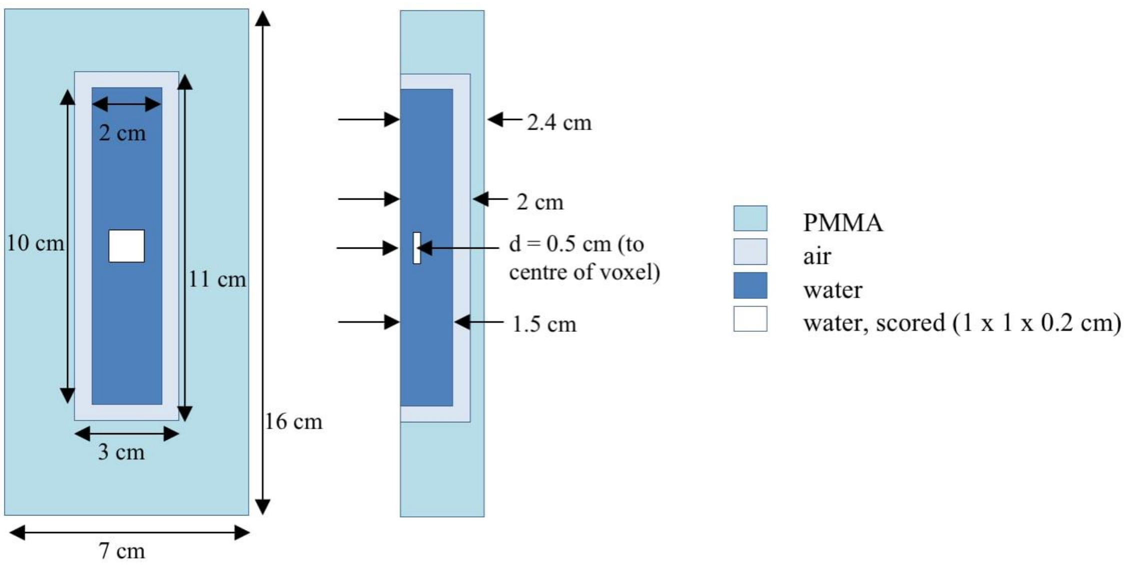

Supplementary Fig. 2. Geometry and composition of the theoretical phantom used to model the mouse and plastic (poly-methyl-methacrylate; PMMA) holder. This phantom was used in the Monte Carlo modelling of the scatter conditions of all three irradiation modalities. While this model does not take into account the exact size or composition of the mouse itself, it does include the overall scatter conditions, and is therefore a more accurate choice than using the dose in a full-scatter water phantom, or the incident air kerma. The geometry of this 'mouse and plastic' phantom has a pronounced effect on MRT valley dose owing to the lack of back-scatter compared to a full-scatter water phantom.

Uncertainty calculation for mouse irradiation dosimetry - CRT

	Value	u (%) Type A	Type B	
**Air kerma rate**				
FC65-G calibration coefficient N_K	4.38 x 10^7 Gy/C		0.70	[1]
chamber-source distance for PBI (TBI)	300 mm (343 mm)		0.50	[2]
Ionization current	1126 pA (833 pA) for 16.5 mA tube current	0.05	0.05	[3]
irradiation non-uniformity PBI (TBI)	1 (0.94)		4.00	[4]
temperature/pressure correction (k_{TP})			0.30	[5]
X-ray stability (no monitor used)			0.30	[6]
possible spectral differences at short distance			0.29	[7]
Quadratic sum		0.05	4.12	
**Combined air kerma rate uncertainty**		4.12		
**Monte Carlo**				
statistical uncertainty		0.30		[8]
differences in geometry/backscatter			4.00	[9]
difference in modelled/actual field size/shape			1.20	[10]
Quadratic sum		0.30	4.18	
**Combined Monte Carlo uncertainty**		4.19		
**Dw uncertainty**				
Air kerma rate		0.05	4.12	
Shutter timing (very small, long exposures)			0.00	
PDD correction to 5 mm depth	0.9966		0.20	
Monte Carlo (field size, mouse holder)		0.30	4.18	
Mass energy attenuation coefficients	1.086		1.5	[11]
Quadratic sum		0.30	6.06	
**Combined D_(w,z=0.5) uncertainty (k=1)**		**6.07**		

$$D_{w,z=0.5} = MN_K \left[\left(\frac{\overline{\mu_{en}}}{\rho}\right)^{w}_{air}\right]_{air} \left[\frac{D_{w,z=0.5}}{D_{w,air}}\right]_{MC}$$

[1] Inherent calibration uncertainty, from ARPANSA calibratkion for air kerma at this beam quality (NXE250)

[2] Approximate 1/r^2 uncertainty in positioning accuracy of 0.5-1mm in 300mm. Also note that SSD was d - 5 mm

[3] Type A is ESDM in typical current measurement, Type B is calculated in worksheet "IC current type B", U8 spreadsheet

[4] Beam non-uniformity for the largest exposed site (measured with film) is a 26% correction with significant asymmetry at one end (down to 40% for TBI).
Estimate uncertainty is 100% of largest correction, which is 6% (DB - I've kept this at 6% but I think its defintion should include more geometry of the mouse)

[5] See U26 - different lab and detector but the estimate is still valid and dominated by possible difference between temperatures at chamber and thermistor

[6] Based on therapy QC history

[7] Guess, chamber NK changes 0.5% with HVL change of ~30%, unlikely HVL will change more than that with distance, treated as rectangular distribution

[8] from Monte Carlo calculation

[9] Monte Carlo calculation based on jig irradiated at centre (actually moved depending on exposed site) and no lead shielding included in model which affects backscatter
Rough calculation based on ratios of irradiated field sizes and TG-61 backscatter factors (which assumes full scatter conditions) gives up to 4% correction for smallest exposed sites (head and thorax, see 'backscatter with lead' tab), estimate uncertainty is 100% of this correction
DB: Even when including the full MC calculation of the dose, there is about a 4% uncertainty due to the air around the mouse. I think this cannot be got rid of.

[10] Estimated difference between FWHM of 9.5 cm and 10.3 cm (approximate measured value and value used in model)

[11] From TG-61

Uncertainty calculation for mouse irradiation dosimetry - SBBR

	Value	u (%) Type A	Type B	
**Absorbed dose to water**				
Pinpoint chamber calibration coefficient $N_{D,w}$	2.70 x 10^9 Gy/C		2.20	[1]
chamber depth in phantom			0.50	[2]
Ionization current		0.05	0.05	[3]
temperature/pressure correction (k_{TP})			0.50	[4]
Beam stability (no monitor used)			0.13	[5]
Interpolation to synchrotron spectrum			0.50	[6]
Field size and distance differences			0.50	[7]
Correction to 5 mm depth			0.00	[8]
Quadratic sum		0.05	2.42	
**Combined dose to water uncertainty**		2.42		
**Monte Carlo**				
statistical uncertainty		0.30		
differences in geometry/backscatter	0.944 for 30x30		4.00	[9]
Cu, Cu BSF correction applied to Mo,Mo			1.00	[10]
difference in modelled/actual field size/shape			0.50	[11]
Quadratic sum		0.30	4.15	
**Combined Monte Carlo uncertainty**		4.16		
**Dw uncertainty**				
Dw rate		0.05	2.42	
Monte Carlo (field size, mouse holder)		0.30	4.15	
Quadratic sum		0.30	4.81	
**Combined D_(w,z=0.5) uncertainty (k=1)**		**4.82**		

$$D_{w,z=0.5} = PDD_{2.00,0.05} M N_{D,w}$$

[1] Inherent calibration uncertainty, from PTB Certificate

[2] ApproximatePDD uncertainty in positioning accuracy of 0.5-1mm in water PDD

[3] Type A is ESDM in typical current measurement, Type B is calculated in worksheet "IC current type B", U8 spreadsheet

[4] TP variation mostly due to possible temperatre and pressure lags. Larger in the synchrotron than ARPANSA.

[5] Synchrotron output variation in top up mode: max 0.5 mA in 200 mA - so 0.25% max (divide by 2)

[6] Estimate of interpolating to Mo/Mo spectrum - kQ from PTB is a slowly varying function with energy

[7] Possible variation due to different fields sizes etc. But this is an ion chamber - insensitive to such things

[8] Ion chamber measurements performed at 5mm depth in soid water. Positional uncertainty accounted for in [2].

[9] A correction is applied to account for the difference between fullscatter conditions and the simplified mouse model.

The uncertainty is 4% due to variable field size due to Pb strips during exp.

[10] An additional 1% since the backscatter correction factor is applicable to the Cu,Cu spectrum only.

[11] The beam is almost parallel when the BDA defines the beam's static height. Small beam divergence accounted for in simulation. Field size variation conservatively estimated to be +- 4mm

Corresponds to a 0.5% change in dose

Uncertainty calculation for mouse irradiation dosimetry -MRT

	u (%) Type A	Type B	
**Absorbed dose to water - peak dose**			
SBBR absorbed dose at 5 mm depth	0.30	4.81	[1]
Output factor OF	0.40	1.69	[2]
Quadratic sum	0.50	5.10	
**Combined dose to water uncertainty**	5.12		

	Type A	Type B	
**Absorbed dose to water - valley dose**			
Absorbed Peak dose at 5 mm depth	0.50	5.10	
PVDR	0.80	6.85	[3]
Quadratic sum	0.94	8.54	
**Combined dose to water uncertainty**	8.59		

Peak dose $D_{w,z=0.5} = \text{OF x } D_{w,z=0.5}$

Valley dose $D_{w,z=0.5} = \text{PVDR x OF x } D_{w,z=0.5}$

[1] Measured for SBBR, including corrections for water cube to mouse model. The interpolation to Mo,Mo uncertainty from SBBR RT [6] is not included since it is measured in Cu,Cu.
Also absent is the additional 1% Mo,Mo uncertainty in the SBBR MC backscatter mouse model correction [9].

[2] The output factor is calculated from MC.
The positional uncertainty is estimated to be 1.64% due to a +- 1mm shift with depth.

The uncertainty due to variation in field size is 0.38% based on a +- 4mm vertical shift.

The uncertainty estimates are combined in quad.

[3] PVDR also calculated using MC. The PVDR is heavily dependent on the depth and field size.

The positional uncertainty is estimated to be 5.9% due to a +- 1mm shift with depth.

The uncertainty due to variation in field size is 3.6% based on a +- 4mm vertical shift

The uncertainty estimates are combined in quad
